# Generation of non-genomic oligonucleotide tag sequences for RNA template-specific PCR

**DOI:** 10.1186/1472-6750-6-31

**Published:** 2006-07-05

**Authors:** Fernando Lopes Pinto, Håkan Svensson, Peter Lindblad

**Affiliations:** 1 Department of Physiological Botany, The Ångström Laboratories, Uppsala University, Box 523, SE-75120, Uppsala, Sweden; 2 Department of Molecular Evolution, Evolutionary Biology Centre, Uppsala University, Norbyvägen 18, SE-75236, Uppsala, Sweden

## Abstract

**Background:**

In order to overcome genomic DNA contamination in transcriptional studies, reverse template-specific polymerase chain reaction, a modification of reverse transcriptase polymerase chain reaction, is used. The possibility of using tags whose sequences are not found in the genome further improves reverse specific polymerase chain reaction experiments. Given the absence of software available to produce genome suitable tags, a simple tool to fulfill such need was developed.

**Results:**

The program was developed in Perl, with separate use of the basic local alignment search tool, making the tool platform independent (known to run on Windows XP and Linux). In order to test the performance of the generated tags, several molecular experiments were performed. The results show that Tagenerator is capable of generating tags with good priming properties, which will deliberately not result in PCR amplification of genomic DNA.

**Conclusion:**

The program Tagenerator is capable of generating tag sequences that combine genome absence with good priming properties for RT-PCR based experiments, circumventing the effects of genomic DNA contamination in an RNA sample.

## Background

Due to its very high sensitivity, reverse transcriptase polymerase chain reaction (RT-PCR) [1] is an extensively used technique for the detection of even very low copy mRNA transcripts. This remarkable sensitivity is also its major shortcoming – RT-PCR is extraordinarily susceptible to DNA contamination. Since PCR is unable to distinguish between cDNA targets and genomic DNA contamination, false positives and/or erroneous quantitative results are possible [2-9].

Ideally, it should be possible to obtain RNA with no DNA contamination at all. Unfortunately, most techniques employed in RNA extraction fail to eliminate all genomic DNA contamination. Assuming that no extraction method can guarantee the absolute absence of DNA in a RNA sample, the ideal RT-PCR procedure should permit the clear distinction between cDNA and contaminating DNA.

Several strategies can be used to overcome DNA contamination [6]. Procedures like oligo d(A) selection, intron spanning primer design, DNase I treatment or restriction endonuclease digestion are standard [2-4]. Use of any of these strategies, or even combinations of them, is common – but can also be time consuming, expensive or can lead to RNA degradation.

In the case of prokaryotes, our main research focus, further limitations exist since oligo d(A) selection and intron spanning primer design are not applicable solutions. On the other hand, the use of anchors, or tags, in the 5' region of a gene specific primer or poly-T tail allows for RNA-specific amplification [7-9], and constitutes a viable strategy. Techniques such as RS-PCR [7] and (EXACT) RT-PCR [9] are based on the integration of such tags (unique sequences not present in genomic DNA) in the 5' end of the first strand cDNA, permitting RNA-specific amplification without loss of sensitivity.

However, the high number of organisms currently used in research results in increased sequence data. As a result of that data increase, tags that were before considered adequate, or are even part of commercially available kits, are now not totally appropriate for use with all organisms. In our opinion, to bridge the potential of these previously described methods with the possibility to use genome-absent tags would give researchers the opportunity to more reliably employ both RS-PCR and (EXACT) RT-PCR for a wider range of organisms.

Given the need to further improve ongoing transcription studies, and the absence of software available to produce RS-PCR suitable tags, it was decided to develop a simple tool that could fulfill such requirements. Tagenerator, the tool presented here, generates genome-absent tags, for RS-PCR and (EXACT) RT-PCR, which constitute good primers during cDNA amplification.

## Implementation

Good primer design is crucial, in order to carry out specific, high yield, PCR reactions. To achieve that, the following tag construction parameters were considered and implemented: tag length, melting temperature, GC content, absence of repeats and absence of secondary structures.

The program Tagenerator is written in Perl [see Additional file 1], requiring the presence of the BioPerl module [10] and a local installation of BLAST [11], from the NCBI toolkit [12]. Options for running the program include the desired tag length, genome/sequence of interest, GC content range and melting temperature range [see Additional file 2]. A compiled version of Tagenerator is also available, for Windows, including usage instructions and requirements list [see Additional file 3].

**Figure 1:**
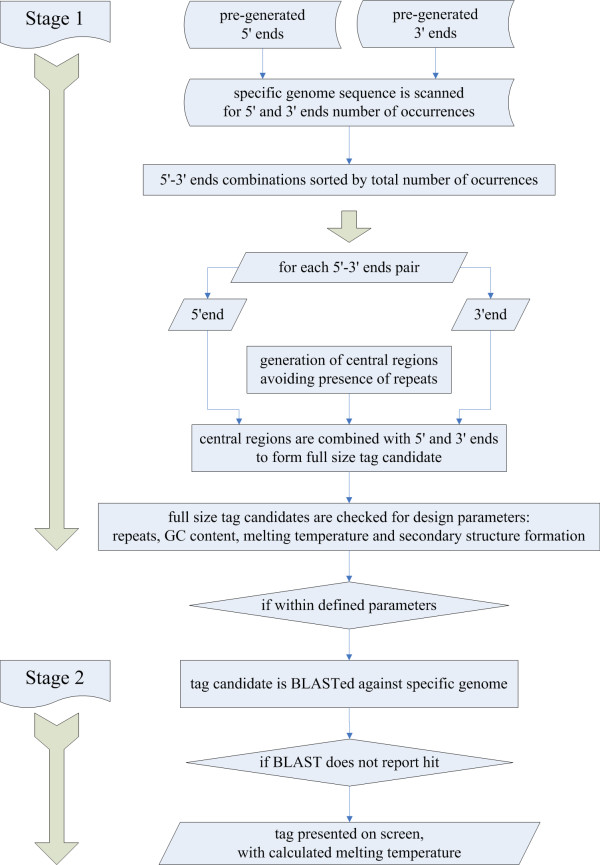
Schematic describing the tag generation and validation process.

The execution comprises, as shown in Figure 1, two main stages: 1) the generation of tag candidates that are good primers and 2) selection for tag candidates that are not present in a given genome (or any other sequence formatted for use with the local BLAST).

Each tag candidate is built in a semi modular fashion, associating a 5' end module, a 3' end module and a random generated central region. Since the 5' ends and the 3' ends are very important for primer quality, these were pre-generated. Two lists of five base long 5' ends and five base long 3' ends were created and integrated in the Perl script. These lists comprise 5' and 3' ends that will have very weak or no interaction, to avoid hairpin formation. In order to increase the overall speed of the process, Tagenerator starts by scanning the sequence of interest for occurrences of all the 5' ends and the 3' ends. Then the program creates a list of all possible 5'-3' ends combinations, sorted by the total number of occurrences.

The central region of the tag candidate is built by combining the four bases. The starting base is random, so that the program is likely to give different tags each time it is invoked. As each base is added, the incomplete sequence is checked for repeats. If an unwanted combination of bases is formed, the last inserted base is replaced. Once a valid full-length intermediate region is obtained, it is associated with the 5' and 3' end forming the full-sized tag candidate.

The complete tag candidate is then examined, so that it complies with the other user defined parameters – GC content is verified followed by the melting temperature. The melting temperature is calculated using nearest-neighbor thermodynamic parameters from SantaLucia *et al *[13], with correction for salt concentration (50 mM Na^+ ^is the assumed default value) according to the work presented by Owczarzy *et al *[14].

After all user defined requirements have been fulfilled, the tag candidate is checked for putative dimer and hairpin formation. Secondary structure formation is evaluated considering the free energy (deltaG) of the interaction for each possible dimer configuration [13]. Only tag candidates for which the maximum free energy is higher than -4 kcal/mol are accepted.

At this point, the tag candidate is blasted against the genome, and if it is found to be present in the genome it is discarded. The BLAST settings defined are *length *7 and an *E value *of 10. With such settings, even statistically poor hits will result in rejection of the tag candidate. If BLAST doesn't report any hits the tag candidate is accepted.

## Results and discussion

### Tags do not amplify genomic DNA

**Figure 2:**
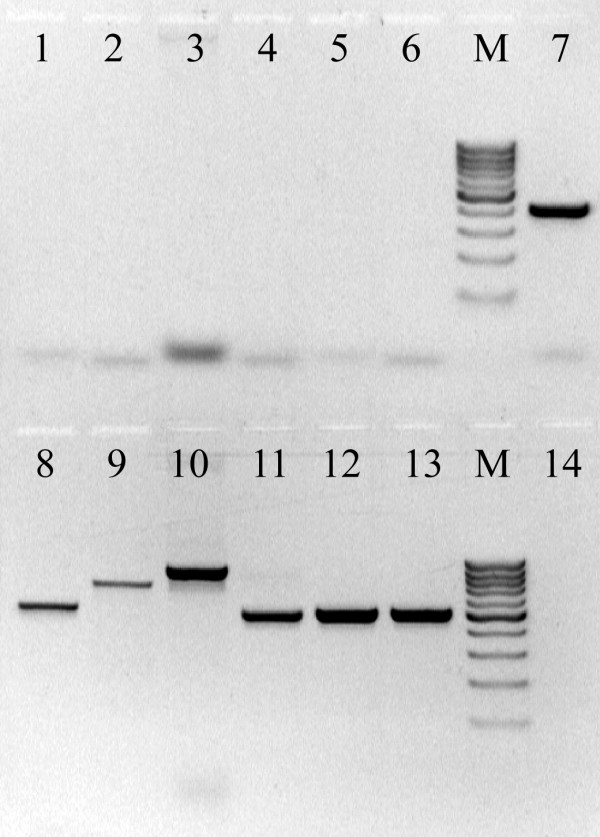
Agarose gel separation of PCR products. Lanes M – molecular weight markers (GeneRuler 100 bp DNA Ladder, Fermentas). Lanes 1 to 6 – PCR reactions using tags for priming and genomic DNA as template (see Table 1). Lane 7 – PCR positive control using a primer pair for *fts*Z and *Nostoc *PCC73102 genomic DNA as template. Lanes 8 to 13 – PCR reactions using genome specific primers and genomic DNA as template (see Table 1). Lane 14 – PCR negative control using a primer pair for *Nostoc *PCC73102 *fts*Z gene.

In order to test the resulting tags, two sets of tests were prepared. The first set of tests concerned: a) the ability of the software to generate tags for a diverse group of organisms (Table 1), with a wide range of genome sizes and b) the possibility to use tags during PCR, having 5 ng of genomic DNA as template, resulting in no amplification (Figure 2).

**Table 1:** Genomes used for tag generation and testing.

**Organism**	**Group**	**tag**	**Lanes in Figure 2**
*Gallus gallus*	Animals	CACTCACAAGCTCGACGTACAC	1 and 8
*Canis familiaris*	Animals	CAGACAGCACTCGTTCGTACAC	2 and 9
*Arabidopsis thaliana*	Plants	GACTGAACGTGCTCTGCTACTG	3 and 10
*Sulfolobus acidocaldarius *str. DSM639	Crenarchaeota	CAGTCACAGCACACGAGTACAC	4 and 11
*Bartonella henselae *str. Houston-1	Alphaproteobacteria	CAGACACGAGCAACGACTACAC	5 and 12
*Bartonella henselae *str. UGA 8	Alphaproteobacteria	CAGACACGAGCAACGACTACAC	6 and 13
*Anabaena *PCC7120	Cyanobacteria	CACTCTGTGCTCGTTGCTACAC	(Figure 3)
*Synechocystis *PCC 6803	Cyanobacteria	CAGACAGCAAGCAGCACTACAC	(Figure 3)

### Tags specifically amplify cDNA

For the second set of tests, RT-PCR was performed having cyanobacterial mRNA as template. For all experiments the reverse transcriptase reaction was performed having a tagged antisense gene specific sequence as primer. The obtained cDNA was then used as template for PCR, using gene specific sequences as forward primers and tags as reverse primers.

For gene *sll1220 *[GenBank: NC_000911 REGION: complement (1678044..1678565)], in *Synechocystis *PCC6803, the following set of primers was used:

tag1220 – CAGACAGCAAGCAGCACTACAC

tagged GSP – CAGACAGCAAGCAGCACTACACCACACAGGTATGTTTCC

sense primer 1220 – CATCTGCGGCCCATCCTA

antisense primer 1220 – TCGCCACTCCAAACACCC

For gene *alr0762 *[GenBank: NC_003272REGION: 883817..884533], in *Anabaena *PCC7120, the following set of primers was used:

tag0762 – CACTCTGTGCTCGTTGCTACAC

tagged GSP – CACTCTGTGCTCGTTGCTACACGAAGTACAAGTGTCAGAG

sense primer 0762 – GGATGGAAGTTCGCACAAATAG

antisense primer 0762 – AAGGTTGGCTGAGGTCGGTA

### Overview of the results

**Figure 3:**
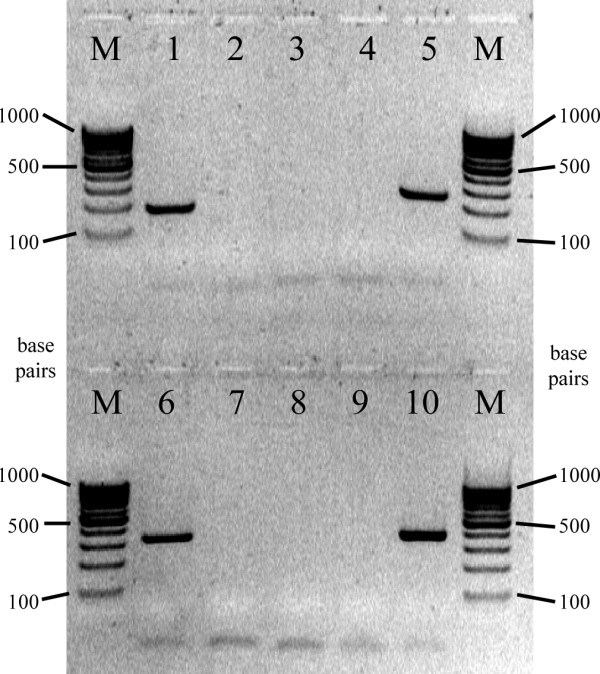
Agarose gel separation of PCR products. Lanes M – molecular weight markers (GeneRuler 100 bp DNA Ladder, Fermentas). Lane 1 – PCR positive control using *sll1220 *sense and antisense primers, and *Synechocystis *PCC6803 genomic DNA as template. Lane 2 – PCR negative control using *sll1220 *sense and antisense primers. Lane 3 – PCR using tag1220 for priming and *Synechocystis *PCC6803 genomic DNA as template. Lane 4 – PCR using tag1220 and sense primer 1220 for priming and *Synechocystis *PCC6803 genomic DNA as template. Lane 5 – PCR using tag1220 and sense primer 1220 for priming and *Synechocystis *PCC6803 tagged cDNA as template. Lane 6 – PCR positive control using *alr0762 *sense and antisense primers, and *Anabaena *PCC7120 genomic DNA as template. Lane 7 – PCR negative control using *alr0762 *sense and antisense primers. Lane 8 – PCR using tag0762 for priming and *Anabaena *PCC7120 genomic DNA as template. Lane 9 – PCR using tag0762 and sense primer 0762 for priming and *Anabaena *PCC7120 genomic DNA as template. Lane 10 – PCR using tag0762 and sense primer 0762 for priming and *Anabaena *PCC7120 tagged cDNA as template.

The assays performed demonstrated that the use of a tag as primer for genomic DNA amplification did not yield any products (Figures 2 and 3). Even when paired with genome specific primers, no PCR products were detected (Figure 3, lanes 4 and 9). On the other hand, cDNA produced using a tagged primer could be amplified when pairing the tag with an opposite sense sequence specific primer (Figure 3, lanes 5 and 10). Our results also show that, when comparing yields, PCR sensitivity was not reduced by the use of tags – the yields of positive controls (Figure 3, lanes 1 and 6) and the cDNA amplifications are similar (Figure 3, lanes 5 and 10).

These results are concordant with the principles of RS-PCR and (EXACT) RT-PCR, and underline the ability of the generated tags to permit the clear distinction between cDNA and contaminating DNA, without sacrificing sensitivity.

### The possibility of having one "universal" tag

Unexpectedly, the output of several runs of Tagenerator resulted in one "universal" tag. In fact, the BLAST sequence alignment of the *Canis familiaris *tag sequence (see Table 1) against GenBank nr database results in no similarity hit and is a unique sequence. However, our concern is that one universal tag might not always be the most adequate for all experiments due to: a) different melting temperatures can be used for PCR, and b) it will not always be possible to combine a gene specific primer with the "universal" tag, due to the formation of secondary structures.

### Benefits of using Tagenerator

Tagenerator allowed us to improve our molecular work, and seems to fill a void in the bioinformatics field, since no other software is known to us that can design such tags. The software has already been used in experiments not documented here, and further application in RACE experiments is now being investigated.

## Conclusion

Tagenerator is capable of generating tags that combine genome absence with good priming properties for RT-PCR based experiments. The use of such tags will deliberately not result in PCR amplification of genomic DNA, permitting the exclusive amplification of cDNA, therefore circumventing the effects of genomic DNA contamination in an RNA sample.

## Availability and requirements

Project name: Tagenerator

Project web page: 

Operating system(s): Platform independent (Windows XP executable also available)

Programming language: Perl

Other requirements: Perl with BioPerl. BLAST from the NCBI Toolkit

License: GNU GPL

## Abbreviations

A,T,G,C – stand for adenine, thymine, guanine and cytosine.

BLAST – basic local alignment search tool.

cDNA – complementary deoxyribonucleic acid.

DNA – deoxyribonucleic acid.

DNaseI – deoxyribonuclease I.

(EXACT) – exclusive amplification of cDNA template.

GSP – gene specific primer.

mRNA – messenger ribonucleic acid.

NCBI – National Center for Biotechnology Information.

PCR – polymerase chain reaction.

RACE – rapid amplification of mRNA ends.

RNA – ribonucleic acid.

RS-PCR – RNA template-specific polymerase chain reaction.

RT-PCR – reverse transcription-polymerase chain reaction.

## Authors' contributions

Fernando Lopes Pinto proposed the building of Tagenerator, in order to overcome issues related to his molecular biology work. He actively participated in: the conception and design of the software, the performing of molecular experiments, result analysis and interpretation, and manuscript writing and revising.

Håkan Svensson had the main role in the design of Tagenerator, and development of the Perl programming. He actively participated in: conception of the software, result analysis, and manuscript writing and revising.

Peter Lindblad was the main person responsible for the establishing of strategies to test and prove the usefulness of Tagenerator. He actively participated in: the planning of molecular experiments, result analysis, and manuscript revising. All the funding, critical evaluation and approval for this project were his exclusive responsibility.

## Supplementary Material

Additional file 1 Script used for the generation of tags and integration of BLAST analysis Click here for file

Additional file 2 Description of the different options for the use of tagenerator Click here for file

Additional file 3 Compressed file containing compiled version of "tagenerator" and instructions for use Click here for file
